# Editorial: Women in health services: Mental health services 2022

**DOI:** 10.3389/frhs.2022.1054214

**Published:** 2022-11-29

**Authors:** Baltica Cabieses, Chiachen Cheng, Alexandra Obach

**Affiliations:** ^1^Programa de Estudios Sociales en Salud, Instituto de Ciencias e Innovación en Medicina (ICM), Facultad de Medicina Clínica Alemana, Universidad del Desarrollo, Santiago, Chile; ^2^Division of Clinical Sciences, Section of Psychiatry, Northern Ontario School of Medicine University, Thunder Bay, ON, Canada

**Keywords:** mental health, women, services, research, health services

As editors of this Research Topic, we are very pleased to present four high-quality manuscripts to showcase the extraordinary work of women researchers around the world who contribute knowledge in mental health systems research. This Research Topic has two goals: firstly, to produce an issue in which those researchers who identify as women might present their work, with focus on early career researchers, and secondly to celebrate research about mental health services.

Promoting women in academia and research is a global challenge. The full potential of women creativity, flexibility and unique contributions are often not realized because women more often than men leave their research careers. Reasons are multiple and complex, yet gender inequities in science continues to exist (1). While progress has been, and continues to be made, in reducing gender inequality in research and academia, change may come about slowly and is subject to significant variation according to country, research field and other factors (1–3). According to the UNESCO Institute of Statistics (UIS) data, less than 30% of the world's researchers are women. Other studies have found that women in STEM fields publish less, are paid less for doing research, and are not promoted as often in their careers compared to men (4). Informed by these gaps, we chose to highlight research conducted by women. We hope to open windows of opportunity that may otherwise not exist for their important work.

The global incidence and severity of mental health disorders has been established, as well as knowledge about pervasively insufficient mental health services available to people who need these services. According to the Global Burden of Disease Study 2010, the economic burden associated with mental disorders exceed those associated with each of four other major categories of noncommunicable disease: diabetes, cardiovascular diseases, chronic respiratory disease, and cancer (5). Major depressive disorder was the second leading cause of years lost to disability (YLD) globally and ranked among the four largest contributors to YLDs in each of the socially diverse regions spanning six continents (6). Anxiety disorders, drug-use disorders, alcohol-use disorders, schizophrenia, bipolar disorders, and persistent depressive disorders also ranked the 20 conditions contributing to the largest global share of YLDs. In the latest Global Burden of Disease Study, this trend continues along with musculoskeletal problems, and cardiovascular diseases (7). Despite this compelling evidence, mental health services are not meeting the clinical quality and quantity of service needs required in every country and region. In all, advances in efforts to alleviate the human and social costs of mental disorders have been both too slow and too few (8).

The paper by Stewart et al. was completed in Canada and focused on the well-being of caregivers of children and youth. The well-being of caregivers has significant implications for healthy development in children and youth. Although caregiver distress is typical, it can become problematic if caregivers have difficulty identifying and responding to their child's needs. Hence, the purpose of this study was to develop and validate an algorithm for identifying caregivers who are at the greatest risk of experiencing distress. Study results indicated that nearly half of the caregivers who were experiencing distress at baseline continued to experience distress at time two. Further, 13.4% of caregivers who were not experiencing distress at the beginning of the study, were indeed experiencing distress at the follow up assessment. This paper addresses a key public health matter that impacts caregivers, children, and youth.

With focus on pediatric chronic pain and resilience, the study by Young et al. was also conducted in Canada. The authors identified risk factors for chronic pain and exploration of how young people negotiate such risk and express resilience. They hypothesized that children and youth with chronic pain would report greater prevalence of mental health disorders than the general population; those demonstrating greater resilience would have less psychiatric comorbidity. They found that female sex, family history, and lower socioeconomic status were associated with chronic pain. Also, psychiatric conditions were more prevalent in chronic pain patients than in the general population. In all, this innovative original paper addresses the need to approach chronic pain from a more holistic mind-body perspective. More research is required to improve quality of health services in this complex global issue.

The manuscript by Singh et al. was an original evaluation based in United States (US). Their study assessed the development and implementation of guidelines proposed by a task force for best practices in delivering Measurement-Based Care (MBC) in Post-Traumatic Stress Disorder (PTSD). Based on the Strategic Action Field Framework for policy implementation research, they found that major barriers to implementation included, the difficulty to establish bottom-up processes, inability to reach the entire field, and limited diversity in the workgroup. Facilitators for guideline implementation included using consensus to make decisions, support provided to workgroup members by national operations partners, and collaboration and mutual respect among workgroup members. This original implementation science study suggests that a hybrid model of implementation in current complex and pandemic contexts provides a process through which frontline workers can inform policy development and implementation.

Finally, the aim of the study conducted by Kristjanson et al. in Canada, was to understand the experiences of perinatal women randomized to the waitlist condition of a randomized controlled trial. This relevant, original study recognizes that pregnant and postpartum women are at a heightened risk for the development or worsening of mental health problems, especially mood and anxiety disorders. In this sense, timely access to mental health support is critical during the perinatal period (spanning pregnancy to 1-year post-partum) to mitigate potential negative impacts on mother and child. As the authors hypothesized, they found the need for timely access to mental health supports during the perinatal period. They offered several recommendations including providing more frequent waitlist status updates, providing more direct access to intermediate interventions, and triaging patients based on clinical need. Research has found in general adult populations, the association between being waitlisted for mental health services with deterioration in mental health. Hence the relevance of this study is substantial as it sheds a new light to this field and challenges health service delivery for perinatal care.

To conclude, it was our honor to be editors of this Research Topic on women researchers in health and mental health services research. We hope readers will enjoy these four selected studies that Frontiers in Health Services have published. Our goal was to be innovative in the way in which women health researchers and mental health services research are understood and interpreted today. This Research Topic exemplifies more holistic and complex approach to ongoing research.

## Author contributions

All authors listed have made a substantial, direct, and intellectual contribution to the work and approved it for publication.

## Conflict of interest

The authors declare that the research was conducted in the absence of any commercial or financial relationships that could be construed as a potential conflict of interest.

## Publisher's note

All claims expressed in this article are solely those of the authors and do not necessarily represent those of their affiliated organizations, or those of the publisher, the editors and the reviewers. Any product that may be evaluated in this article, or claim that may be made by its manufacturer, is not guaranteed or endorsed by the publisher.

## References

[1] League of European Research Universities (LERU). Women, Research and Universities: Excellence Without Gender Bias. LERU Gender Working Group (2012). Available online at: https://www.leru.org/files/Women-Research-and-Universities-Excellence-without-Gender-Bias-Full-paper.pdf (accessed September 25, 2022).

[2] McKinsey & Company. The Power of Parity: Advancing Women's Equality in Africa. McKinsey Global Institute (MGI) (2019). Available online at: https://www.mckinsey.com/ /media/mckinsey/featured%20insights/gender%20equality/the%20power%20of%20parity%20advancing%20womens%20equality%20in%20africa/mgi-the-power-of-parity%20advancing%20womens%20equality%20in%20africa.pdf (accessed September 25, 2022).

[3] European Commission Directorate-General for Research Innovation. She Figures 2021: Gender in Research and Innovation: Statistics and Indicators. (2021). Available online at: https://data.europa.eu/doi/10.2777/06090 (accessed September 25, 2022).

[4] Unesco Institute for Statistics, UIS. Women in Science. (2019). Available online at: http://uis.unesco.org/en/topic/women-science?wbdisable=true (accessed September 25, 2022).

[5] Bloom DE Cafiero ET Jane-Llopis E. The Global Economic Burden of Non-Communicable Diseases. Geneva: World Economic Forum (2011). Available online at: https://cdn1.sph.harvard.edu/wp-content/uploads/sites/1288/2013/10/PGDA_WP_75.pdf (accessed September 25, 2022).

[6] Vos T Flaxman AD Naghavi M Lozano R Michaud C Ezzati M. Years lived with disability (YLDs) for 1160 sequelae of 289 diseases and injuries 1990-2010: a systematic analysis for the Global Burden of Disease Study 2010. Lancet. (2012) 380:2163–96. 10.1016/S0140-6736(12)61729-2 23245607 PMC6350784

[7] GBD 2019 Viewpoint Collaborators. Five insights from the global burden of disease study 2019. Lancet. (2020). 396:1135–59. 10.1016/S0140-6736(20)31404-5 33069324 PMC7116361

[8] Becker A Kleinman A. Mental health and the global agenda. N Engl J Med. (2013) 369:66–73. 10.1056/NEJMra1110827 23822778

